# Metallic Nanoparticles for the Modulation of Tumor Microenvironment; A New Horizon

**DOI:** 10.3389/fbioe.2022.847433

**Published:** 2022-02-16

**Authors:** Siavash Shariatzadeh, Negin Moghimi, Farima Khalafi, Sepehr Shafiee, Mohsen Mehrabi, Saba Ilkhani, Foad Tosan, Pooria Nakhaei, Ali Alizadeh, Rajender S. Varma, Mohammad Taheri

**Affiliations:** ^1^ Department of Pharmacology, School of Medicine, Shahid Beheshti University of Medical Sciences, Tehran, Iran; ^2^ Department of Anatomy, School of Medicine, Shahid Beheshti University of Medical Sciences, Tehran, Iran; ^3^ Department of Medical Nanotechnology, School of Medicine, Shahroud University of Medical Sciences, Shahroud, Iran; ^4^ Department of Biology and Anatomical Sciences, School of Medicine, Shahid Beheshti University, Tehran, Iran; ^5^ Semnan University of Medical Sciences Dental Student Research Committee, Semnan, Iran; ^6^ School of Medicine, Tehran University of Medical Sciences, Tehran, Iran; ^7^ Deputy of Research and Technology, Ministry of Health and Medical Education, Tehran, Iran; ^8^ Regional Centre of Advanced Technologies and Materials, Czech Advanced Technology and Research Institute, Palacký University in Olomouc, Olomouc, Czech Republic; ^9^ Skull Base Research Center, Loghmna Hakim Hospital, Shahid Beheshti University of Medical Sciences, Tehran, Iran; ^10^ Institute of Human Genetics, Jena University Hospital, Jena, Germany

**Keywords:** microenvironment, gold nanoparticles, silver nanoparticles, metallic nanoparticles, magnetic nanoparticles, cancer

## Abstract

Cancer is one of the most critical human challenges which endangers many people’s lives every year with enormous direct and indirect costs worldwide. Unfortunately, despite many advanced treatments used in cancer clinics today, the treatments are deficiently encumbered with many side effects often encountered by clinicians while deploying general methods such as chemotherapy, radiotherapy, surgery, or a combination thereof. Due to their low clinical efficacy, numerous side effects, higher economic costs, and relatively poor acceptance by patients, researchers are striving to find better alternatives for treating this life-threatening complication. As a result, Metal nanoparticles (Metal NPs) have been developed for nearly 2 decades due to their important therapeutic properties. Nanoparticles are quite close in size to biological molecules and can easily penetrate into the cell, so one of the goals of nanotechnology is to mount molecules and drugs on nanoparticles and transfer them to the cell. These NPs are effective as multifunctional nanoplatforms for cancer treatment. They have an advantage over routine drugs in delivering anticancer drugs to a specific location. However, targeting cancer sites while performing anti-cancer treatment can be effective in improving the disease and reducing its complications. Among these, the usage of these nanoparticles (NPs) in photodynamic therapy and sonodynamic therapy are notable. Herein, this review is aimed at investigating the effect and appliances of Metal NPs in the modulation tumor microenvironment which bodes well for the utilization of vast and emerging nanomaterial resources.

## Introduction

Cancer is a significant concern in modern societies worldwide. Abnormal cell growth and their transformation into different types of tumors in human organs cause this life-threatening complication (Zaorsky et al., 2017; Shahab et al., 2018; DeSantis et al., 2019). Several pathways and molecular defects are significant in the development and progression of cancer. The leading cause of tumor formation is due to one or a series of gene mutations. Moreover, tumor deterioration, benign or malignant tumor, and metastatic behavior depends on the mutation type and affected genes. The tumor stage is the single most determining parameter to select the therapeutic approaches (Paul and Lal, 2017; Mohd Yusof et al., 2018; Zhao et al., 2020).

Surgery, radiotherapy, chemotherapy, or combination therapy are employed as the therapeutic procedure for most of the cancer types (Temple et al., 2004; Giordano et al., 2005; Verbrugge et al., 2009). Today, blended methods are commonly used in combination with chemotherapeutic agents for most cancers treatment, largely trying to control angiogenic, signaling, DNA replication, and cell cycle process (Urruticoechea et al., 2010; Yuan et al., 2019). Despite mentioned advances, cancer therapy is still complicated and sometimes impossible in most cases, especially in the metastatic stages thus necessitating search for new treatments.

The use of NPs has garnered much attention lately as they can be very effective in medicine due to their unique properties, suitable and tunable features for drug delivery, and their effects on the treatment process. To optimize loading and delivery capacity, NP parameters like as shape, size, and surface chemistry have been carefully designed (Singh et al., 2019). One of the essential properties of NPs is the high surface-to-volume ratio, which elevates their surface energy that can be exploited in various medical applications, especially photodynamictherapy (PDT) (Singh et al., 2020). Daniel and Astrum (Daniel and Astruc, 2004) have summarized the history of the nanoparticles and mentioned the use of metal nanoparticles historically and reported the popularity due to uniform size and sharp size distribution. They have provided various fascinating properties leading to remove barriers in different field of nanotechnology specially in biomedical field because of their unique physiochemical properties (Sintov et al., 2016; Venkatesh et al., 2018). Metal nanoparticles which are traditionally are known with silver and gold, provides unique chrematistics such as SPR (surface plasmon resonance) more effectively that other types of nanoparticles. It has been confirmed that rational design of metal nanoparticles represented well biocompatibility and versatility (Patra et al., 2018). Meanwhile, metal nanoparticles can be engineered as theranostics particles to provide both therapy and imaging simultaneously (Sintov et al., 2016). Theranostics based on nanoparticles (NPs) is a promising paradigm in nanomedicine (Singh et al., 2017). The deployment of nanotechnology in the right situation can overcome many challenges in cancer treatment (Alvarez et al., 2017; Maddela et al., 2021). The proper position in cancer treatment is to identify the vulnerability of cancer cells and destroy them without damaging normal cells and tissues. The tumor microenvironment (TME) in many cancers is now considered a critical target for therapy and has been ascribed to as a crucial involved parameter for promoting tumor growth, proliferation, angiogenesis, invasiveness, and metastasis (Liu et al., 2018a).

Mesenchymal cells, extracellular matrix (ECM), cancer-associated fibroblasts (CAFs), and immune system cells are important components of TME in cancer fate and progression to metastasis (Anton and Glod, 2016; Nadhan et al., 2020a). In the early stages of TME formation, cells and proteins involved in cell death are disrupted through an interaction, and the process of cell proliferation and differentiation is affected (Phan, 2008; Yuan et al., 2016; Murphy and Weaver, 2017a; Farc and Cristea, 2021). Proteins involved in the process of reproduction and programmed death include growth factors and inflammatory factors of the immune system that are involved in tumor angiogenesis (Chang et al., 2002; Phan, 2008; Dumont et al., 2013; Yuan et al., 2016). Out-of-regulation function of immune system cells inhibits their function and reduces suppression of tumorigenesis (Samstein et al., 2012; Lei et al., 2020). By reducing the function of immune cells in addition to reducing the immunogenic function of lymphocytes, increasing inflammatory factors such as cytokines and chemokines cause more tumor metastasis (Talmadge and Gabrilovich, 2013; Wolf et al., 2015; Zhou et al., 2018a).

Due to the prominent effect of TME on the proliferation, migration and metastasis of cancer cells, targeting it can be effective in reducing tumor progression (Labani-Motlagh et al., 2020a; Liu et al., 2020). Due to the fact that the effect of MNPs on TME has been rarely studied, so their study can be effective in further understanding these nanoparticles. In the present study, we first identify TME and its characteristics. Then, nanoparticles and their effects on TME and treatments were investigated.

## Tumor Microenvironment

**FIGURE 1:**
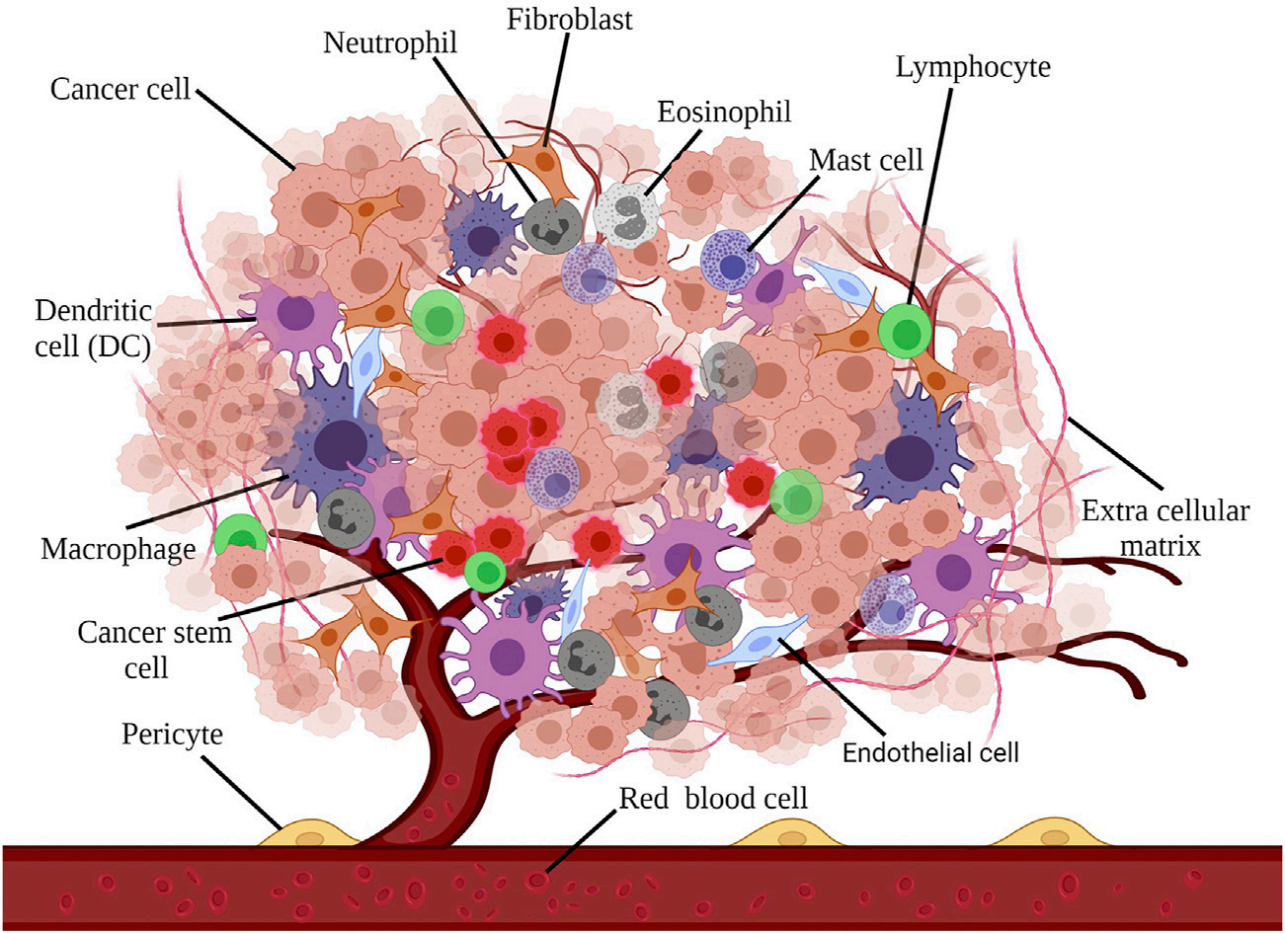
Different cell types are present in TME: macrophages, fibroblasts, endothelial cells, neutrophils, eosinophils, mast cells, lymphocytes, dendritic cells, and dendritic cells, each representing a diverse impact on cancer tissue.

Cancer tissue has a supportive environment in which various components can infiltrate as homeostasis, fighting, or helping elements. TME can involve many cancer processes such as tumor growth, proliferation, angiogenesis, invasiveness, and metastasis via interaction with cancer cells as a dynamic cellular environment. Mesenchymal cells and the extracellular matrix (ECM) as the components of the TME are responsible for secreting various factors which affect cancer fate. Cancer-associated fibroblasts (CAFs) are known to be the main cellular components of TME, which by secreting multiple factors including EGF (endothelial growth factor), VEGF (vascular endothelial growth factor), and HGF (hepatocyte growth factor), can metastasize cancer cells by disrupting and rupturing the ECM via the RTK signal pathway (Anton et al., 2017; Nadhan et al., 2020b). Different cell types are present in TME (Figure 1), Such interaction of cells leads to establishing a complex network that can promote or inhibit cancer reliant on tumor condition and cell interaction (Phan, 2008; Murphy and Weaver, 2017b; Farc and Cristea, 2021). Reprograming the surrounding cells, mostly fibroblasts, immune cells, and vascular cells by tumor cells, is the first step for TME formation (Yuan et al., 2016).

Fibroblasts constitute the main component of the tumor stroma which can be recognized through distinctive markers such as vimentin, smooth muscle actin-α (SMAα), fibroblast activation protein (FAP) (Chang et al., 2002; Phan, 2008). To create CAFs, tumor cells induce fibroblasts and blood vessels by different factors such as PDGF (platelet-derived growth factor) or FGF (fibroblast growth factor) and hypoxia through activating PDGF, IL1, stromal cell-derived factor (SDF), TGFβ, and reactive oxygen species (ROS). Thus, CAFs formed the overall shape of TME through ECM secretion and cytokines and growth factors activating such as TGFβ, HGF, SDF, and MMPs, and inducing angiogenesis by VEGF and PDGF (Dumont et al., 2013).

Immune-related cells are the other most dominant cells in TME. It has been reported that both tumor-antagonizing and tumor-promoting cells are present in TME. Macrophages, neutrophils, natural killer cells, T cells, and dendritic cells are the essential tumor-antagonizing cells of the immune system, while myeloid-derived suppressor cells (MDSCs) and regulatory T cells (Tregs) are among the most significant tumor-promoting immune cells (Lei et al., 2020). Foxp3 is the distinctive marker of Tregs, which is essential for their function (Samstein et al., 2012). Besides the suppressive role, Tregs represent a regulatory function on effector T cells which is significant in some severe conditions, including autoimmune disease that suppresses the over-reactive immune response. Therefore, cancer suppression by cytotoxic T cells possibly can be inhibited due to the presence of Tregs in TME (Wolf et al., 2015). On the other hand, MDSCs use a different mechanism for tumor promotion; they are seemingly induced in TME followed by cytokines and chemokines secretions. Thus, MDSCs exert their impact through cancer cell migration, promote metastasis and angiogenesis (Talmadge and Gabrilovich, 2013; Zhou et al., 2018b).

## TME Modulation

**FIGURE 2:**
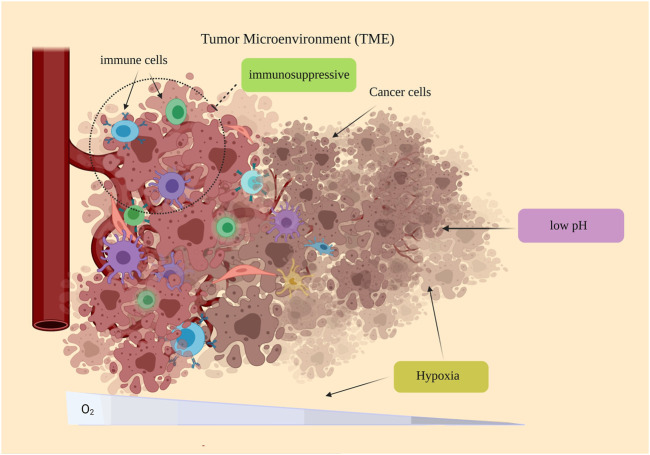
Some special features of the TME: hypoxia, low pH, and immunosuppressive environment. The immunosuppressive environment of TME has been observed in many cancers. Because of rapid proliferation and the imbalance between oxygen supply and consumption, the TME oxygen level tends to be reduced, which is ascribed as hypoxic regions observed in most tumors. PH reduction is another feature of TME.

TME has a substantial effect on metastasis and cancer resistance, so that it is introduced as the primary barrier against the clinical use of immunotherapy. Furthermore, in view of its prominent effect in proliferation, migration, and metastasis, it can be considered an extraordinary target in the treatment of cancer (Liu et al., 2020; Labani-Motlagh et al., 2020b). Given the challenges and limitations of current therapies, TME modulation can be regarded as an alternative approach, which can significantly enhance the effectiveness of existing treatments. In addition, TME represents some unique features, including hypoxia, low pH, and immunosuppressive environment that can be recruited as the target for TME modulation (Figure 2) (Chen et al., 2015; Janoniene et al., 2017; Mpekris et al., 2017).

### TME and Hypoxia

One of the essential elements for energy metabolism is oxygen. Hypoxia causes intratumorally oxygen gradients and increase the hypoxia-inducible factor 1α (HIF-1α), a key marker in hypoxia mechanisms and the central mediator of hypoxia-induced signaling. On the other hand, hypoxic TME leads to tumor development and drug resistance through uncharacteristic angiogenesis, desmoplasia, and inflammation (Mayer et al., 2008; Jain, 2014; Whatcott et al., 2015). HIF-1α has been reported to be overexpressed in various cancer types and has accounted for tumor survival through drug resistance (Sun et al., 2007; Mayer et al., 2008; Simiantonaki et al., 2008). The effect of HIF has been proven in cancer stem cell (Emami Nejad et al., 2021). Besides, hypoxic TME is apparently involved in p53 and mitochondrial regulation and modulation in cancer cells (Jing et al., 2019).

### TME, Low pH and Immunosuppressive Environment

Nevertheless, a low pH environment can be induced by hypoxia in TME, leading to multidrug resistance. Multiple mechanisms have been attributed to this cause, such as genetic alteration, ion trapping, or multidrug transporter p-glycoprotein (P-gp) overactivity. Due to the semi-permeable properties of the cellular membrane, unlike the charged particles, small uncharged molecules can diffuse into the cells (Zub et al., 2015). Considering the pH-depending property of several chemotherapeutic agents, intracellular pH alteration leads to low diffusion of chemotherapies into the cell membrane, resulting in drug resistance. On the other hand, as mentioned P-gp is an essential drug-resistant mechanism in low pH TME (Triner and Shah, 2016).

Considering mentioned properties, treatments based on targeting acidic conditions and hypoxia TME can be very effective. As an example, one approach could target hypoxia by inhibiting VEGEF or PI3K/AKT/HIF-1α pathway (Jing et al., 2019). In general, accurate targeting of one of the main features of TME and its disruption, including hypoxic conditions that subsequently cause high acidity of this structure, can be considered a critical therapeutic approach. However, one of the main problems in this area is the accurate delivery and targeting, which possibly can be overcome with nanotechnology.

## Effect of NPs on TME Modulation

TME modulation, as described in the previous section, can be exploited as one of the main treatments for various types of solid cancers. In this section, the effects of nanotechnology and NPs on TME modulation are highlighted.

NPs are generally referred to as particles with dimensions of about 1–100 nm and possess various attributes and different properties than their bulk sample. Such difference creates very distinctive properties that are generally ascribed to their high surface-to-volume ratio (Maddela et al., 2021). The antibacterial properties of silver NPs have long been recognized (Mozafari et al., 2021). However, in the last few decades, due to the availability of new technologies, it has become possible to make a wide range of NPs, from quantum dot nanoparticles to nanofibers.

The use of NPs for targeted drug delivery has been studied in many diseases, and excellent clinical results have been observed (Tietze et al., 2013; Mu et al., 2020). Due to the NPs’s size and high surface-to-volume ratio, it is possible to load the drug in different parts of NPs, including the surface of the NPs, inside the capsule-like structures and connecting to internal components such as those seen in dendrimers. Therefore, they can provide effective TME modulation (Dadwal et al., 2018; Hu et al., 2019). One of the critical drug accumulators in cancer is the enhanced permeability and retention (EPR) effect. It has been well understood that NPs (20–200 nm) can effectively get accumulated in cancer cells due to their adoptable size to the vascular endothelial pores and permeability (Huai et al., 2019).

Despite the unique opportunities that EPR plays in NPs accumulation in cancer cells, studies have shown that due to the high heterogeneity of cancer cells and the abnormality of vasculature in cancer tissue, NPs did not reach the cancer tissue more effectively. Therefore, researchers have resorted to active-targeted drug delivery approaches because the EPR effect in drug delivery systems is considered passive. In this regard, NPs are designed to target specific cancer markers. Due to the extremely high cancer antigenicity, one of the factors that are mainly considered in the active targeting of cancer cells is the VEGF receptor. Consequently, the design of NPs based on this factor and the simultaneous use of the EPR effect can increase the drug accumulation in TME to an adequate level. Among other cancer-targeting agents, anginex and RGD peptides can be mentioned for targeting galectin-1 and integrin αvβ3, respectively (Byrne et al., 2008; Zhu et al., 2017; Liu et al., 2018b; Fu et al., 2019).

Moreover, metal NPs can be considered as a therapeutic agent in addition to being drug delivery entities. In this regard, plasmon resonance and photoluminescence properties of metal NPs can be mentioned (Hu et al., 2019). Furthermore, NPs have been used in various studies for TME modulation as follows: for modulation of the acidic TME, modulation of tumor ECM structure, immunosuppressive TME modulation, and also for the reduction of tumor hypoxia by oxygen delivery, oxygenation, and alleviate oxygen consumption (Liu et al., 2018a).

## Metal Nanoparticles for TME Modulation

One of the most attractive NPs in the field of biomedicine and especially drug delivery are metal NPs. These NPs, which are generally between 1 and 100 nm in size, have extraordinary properties that distinguish them from other NPs. These unique properties include magnetic, optical, and catalytic properties. Metal NPs have various capabilities depending on the type, material, shape, composition, and size. With the precise engineering of these NPs, one can expect to receive multiple responses under the same conditions. Also, with the accurate design of Metal NPs in terms of composition and size, the bioavailability, biological activity, and toxicity, as one of the central Metal NPs limitations, can be controlled (Sharma et al., 2018).

**FIGURE 3:**
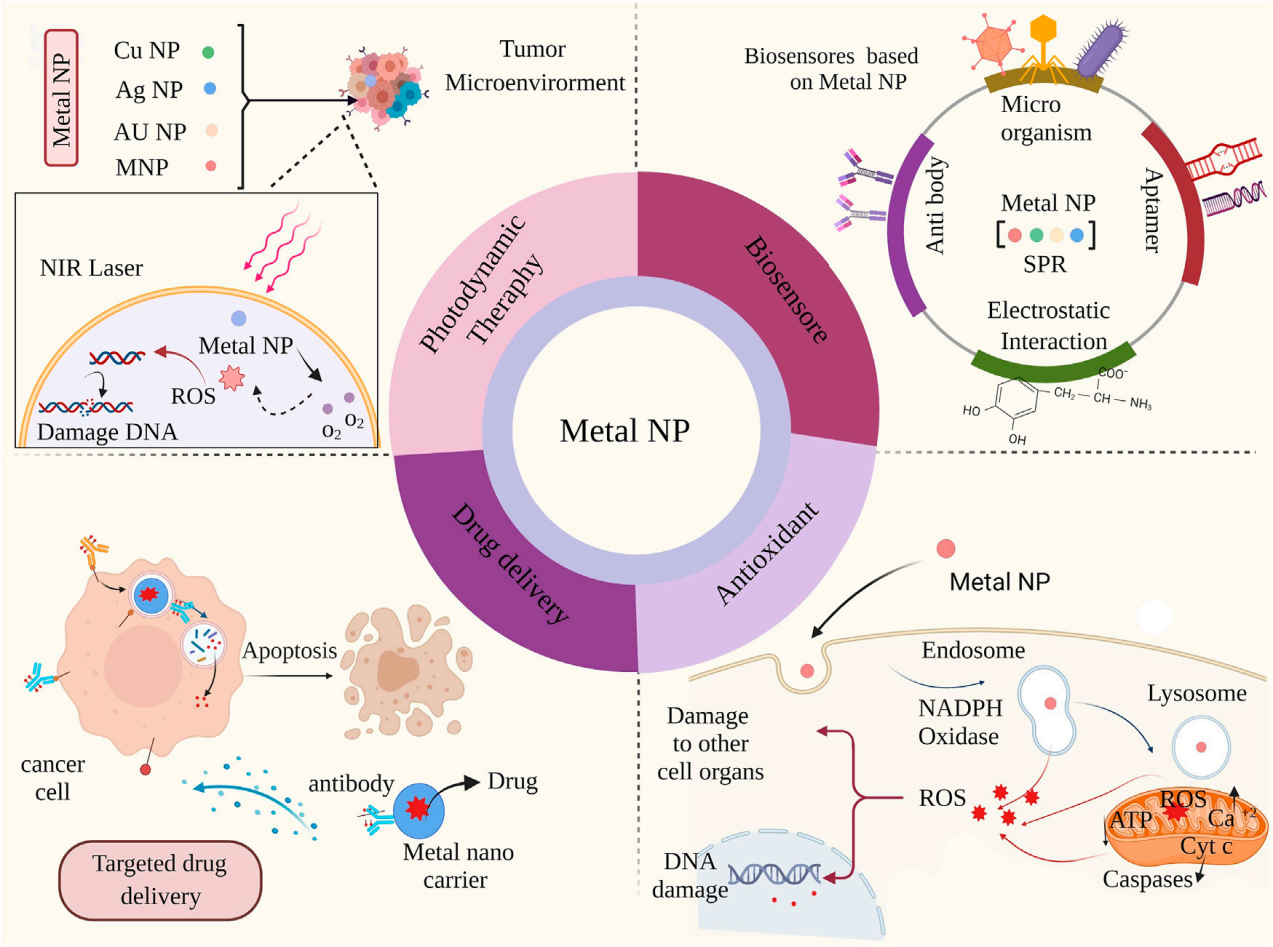
Miscellaneous biomedical applications of metal nanoparticles: Metal nanoparticles cause further damage to cancer cells and cell death through photodynamic treatment with TME irradiation. Metal nanoparticles can also be used as biosensors.

Due to the unique properties, Metal NPs are widely studied in cancer therapy through various approaches, including drug delivery, PDT, and antioxidants. However, one of the essential properties of Metal NPs, especially gold, silver, and copper nanoparticles, is the presence of surface plasmon resonance (SPR); SPR refers to the oscillation resonance of surface electrons in particles that are excited by light. Because nanoparticles have a larger surface-to-volume ratio compared to their bulk particles, SPR will be much more pronounced in them. Therefore, the SPR property has been widely used in photodynamic therapy through nanoparticles. Specifically, individual nanoparticles can be designed to be responsive to near-infrared absorbance to acquire photothermal agents to treat the tumor (Bhattacharyya et al., 2011). Furthermore, gold-based nanostructures, rhodium, and CuS nanoparticles have been proposed to provide photothermal responses, which can be recruited as biosensors despite the therapy approach. Considering all these properties and simultaneous use as a drug delivery system, Metal NPs appear to be an ideal option for TME modulation (Figure 3).

Metal NP -based sensors can lead to significant signal amplification, higher sensitivity, and great improvements in the detection and quantification of biomolecules and different ions. Nanoparticles with antioxidant properties increase cell damage by increasing cellular ROS (Sharma et al., 2018; Fu et al., 2019). ROS are group of materials including H_2_O_2_ and hydroxyl radicals (·OH) generated in eukaryotic cells. Despite previous thought that considered ROS as the byproduct of cells, it has been confirmed that ROSs are involved in several signaling pathways. ROS are produced in mitochondria through reduction of oxygen molecules form superoxide anions, peroxisomes and the endoplasmic reticulum. ROS are essential for multiple cellular functions at the normal level, such as gene expression (Chakraborty et al., 2021; Grebinyk et al., 2021). However, excess ROS production have been documented in various tumor cells due to increased metabolic rate, gene mutation and relative hypoxia (Wilson et al., 2018). The overload ROS can damage the normal cell causing various pathological conditions (Xiao et al., 2012a; Shanmugam et al., 2014a). Hence, the ROS modulation can be an appropriate approach regarding cancer treatment. cerium dioxide nanoparticles (CeNPs) is an almost new emerging nanoparticle regarding cancer treatment via ROS modulation (Shanmugam et al., 2014b). CeNPs have presented a powerful redox property through witching the oxidation state of Ce3+ and Ce4+ (Wang et al., 2009; Tsai et al., 2013). According to previous studies CeNPs can modulate ROS state through catalase- and superoxide dismutase (SOD)-like activity (Choi et al., 2007; Xiong et al., 2020). Filippi et al. have reported that CeNPs exert high ·OH scavenging activity in both phosphate buffered saline and surrogate lung fluid (Shanmugam et al., 2014b). In drug delivery, they cause programmed death by entering cancer cells (Bhattacharyya et al., 2011).

### Metal NPs for Modulation TME Hypoxia

Hypoxia, low oxygen, and oxygen overconsumption is a significant feature of TME. Therefore, modulation of TME hypoxia appears to be a practical approach for tumor treatment. One system to modulate TME is related to oxygenation which is most often used during photodynamic therapy. Oxygen molecules can produce highly stable peroxides that bind to the broken ends of DNA, which dramatically enhances photodynamic therapy. It can be acknowledged that the oxygen molecule has a dual behavior in the treatment of cancer.

On the one hand, by increasing the amount of oxygen in the TME, ionizing radiation produces free radicals that can destroy DNA beyond repair. On the other hand, with the lack of oxygen, the effect of ionizing radiation on photodynamic therapy on the breakdown of DNA dual strands seems to be seriously reduced (Yoshimura et al., 2013). In this context, Metal NPs can function well as photosynthesizers and significantly increase the effect of radiation on TME modulation.

One of the most exciting nanoparticles in photodynamic therapy as photosynthesizers due to their catalytic properties, is gold nanoparticles. The effect of gold nanoparticles is due to their high energy transfer in the excited state to molecules such as oxygen. In this regard, it causes highly toxic ROS species that modulate TME and eventually kill the cancer cell mass. Due to this mechanism, the presence of molecules such as O_2_ can significantly increase the ability of nanoparticles to produce ROS, including ^1^O_2;_ it also prevents the PDT from being endangered due to the hypoxic state of TME (Dhakshinamoorthy et al., 2020; Yang et al., 2021).

Liang et al. have used gold nanocages@manganese dioxide to impede tumor metastasis through PDT and oxygenation. First, they fabricated core-shell anocage@manganese dioxide (AuNC@MnO_2_, AM) nanoparticles using the template method. Next, a laser instrument has been recruited to induce PDT of this nanoparticle. The mechanism underlying oxygen generation was due to the degradation MnO_2_ part in the low pH microenvironment of cancer, which leads to a large amount of production of O_2_, which finally significantly enhanced the PDT effect on breast cancer cell line (Liang et al., 2018). In another study, wang et al. have reported the benefit of a rhodium-gold metals-based porous core-shell nanoparticle-elevated oxygenation to promote PDT for cancer therapy (Wang et al., 2020a).

**FIGURE 4:**
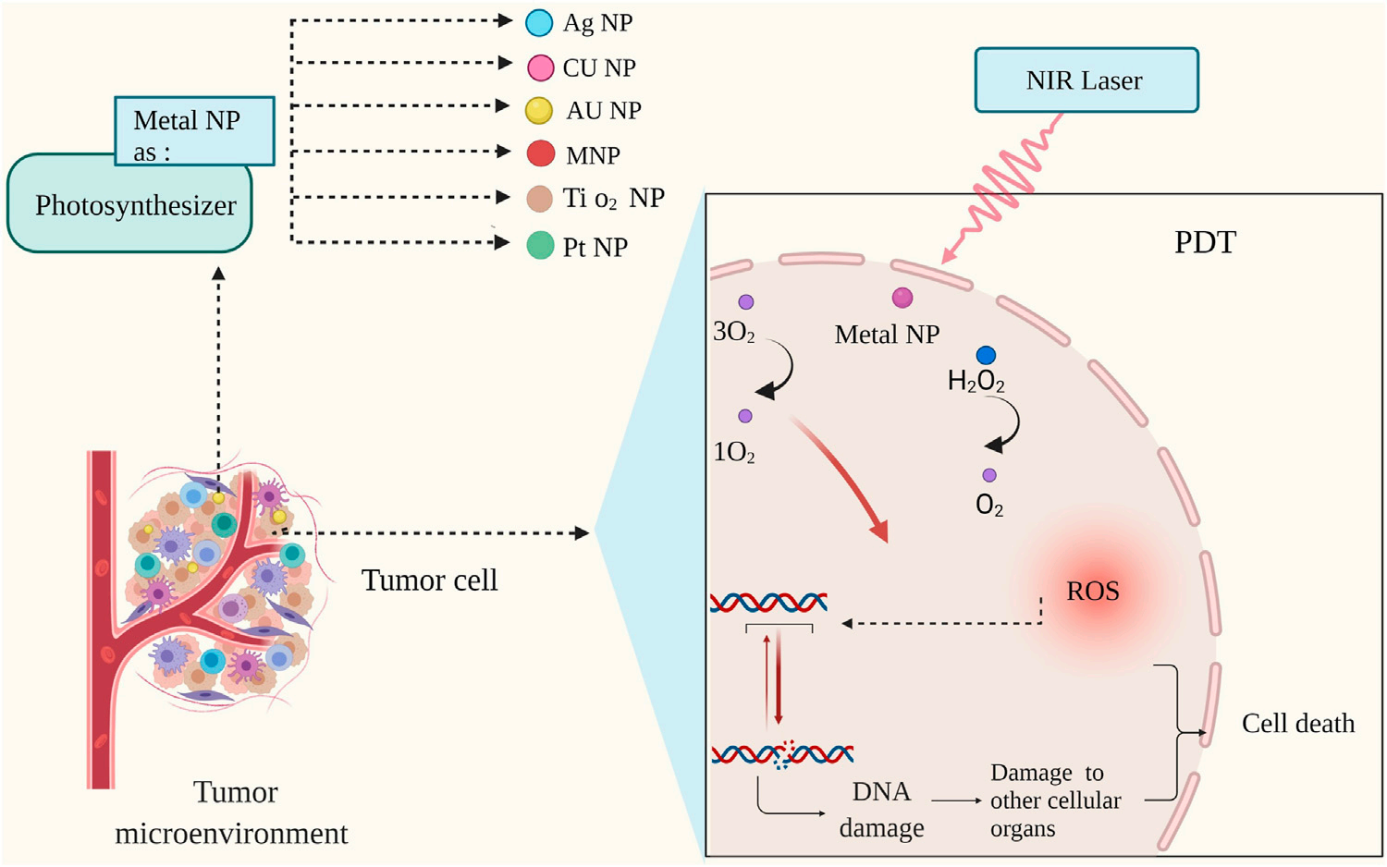
Mechanism of photodynamic therapy (PDT) with metal nanoparticles. During photodynamic therapy, nanoparticles increase oxygen production; This increase in cell oxygen production is associated with damage to nucleic acid, and the cascade created by the nanoparticles leads tumor cells to planned death (Liang et al., 2018; Yang et al., 2020b).

As mentioned earlier, TME hypoxia itself is a barrier to successful PDT. However, developing hypoxia in TME using other systems is a treatment procedure. For example, sonosensitizers is a substance that reacts with ultrasound waves to produce ROS in cancer cells (Liang et al., 2018; Wang et al., 2020b). However, one of the problems with organic sonosensitizers is their stability and low solubility in aqueous media. For this reason, much attention has recently been paid to develop inorganic sonosensitizers, comprising Metal NPs (Figure 4).

Titanium dioxide nanoparticles, for example, can serve as a sonosensitizer on their own (Pan et al., 2018; Yang et al., 2020a). Zhong et al. developed a type of sonosensitizer using copper metal where copper divalent ions cause GSH depletion through the redox reaction, which ultimately increases the hypoxia of cancer cells. This group used the Pt and Cu elements to fabricate PtCu_3_ PtCu_3_ nanocage sonosensitizer via solvothermal method. Furthermore, they pegylated the nanocage that induced peroxidase activity wherein pegylated PtCu3 nanocages could act as glutathione peroxidase, accelerating the process of GSH depletion in the presence of oxidase molecules. Additionally, their anticancer effect was examined both, *in vitro* and *in vitro* in cancer mice models. The results showed that these metal nanosonosensitizer with ultrasound had the excellent effect on killing cancer cells (Zhong et al., 2020).

### Metal Nanoparticles for TME Modulation Using Low pH

Another exciting feature of TME is its high pH compared to normal cells due to the increased metabolism of cancer cells. This feature has been used in many cancer treatment approaches (Barar and Omidi, 2013; Justus et al., 2013). Zhang et al. have proposed a pH-responsive loaded-doxorubicin (DOX) metal-organic framework (MOF, ZIF-8) gold nanocluster (AuNCs@MOF-DOX) for modulation of the breast cancer TME as a PDT/chemotherapy combination therapy; both, the AuNCs and DOX are released through ZIF-8 collapse due to the low pH condition in TME. Next, AuNCs and DOX serve as the PDT and chemotherapy agents, representing significant cancer cell killing compared to a single action (Zhang et al., 2020).

One effective nanoparticle subdivision for TME modulation is attributed to the ferromagnetic nanoparticles (γ-Fe_2_O_3_ or Fe_3_O_4_ NPs). In normal cells, they transform the toxic H_2_O_2_ into H_2_O and O_2_, while in the low pH condition of TME, ferromagnetic can catalytically produce highly toxic ROS such as hydroxyl radicals (·OH) from H_2_O_2_ (Huai et al., 2019). In addition, Fu et al. have investigated the effect of the different structures of Fe_3_O_4_ on cancer therapy. The intrinsic peroxidase-like activity of Fe_3_O_4_ has been well established as various designs, including nanoclusters, nanoflowers, and nanodiamonds, were used to analyze the peroxidase activity of Fe_3_O_4_ in the low pH of the cancer microenvironment. According to their *in vitro* evaluation, the nanoclusters form had the most critical effect on the peroxidase-like activity of Fe_3_O_4_ NPs. They also reported that the cancer cell death followed by Fe_3_O_4_ could be attributed to the ROS generation just after the endocytose and concluded that cancer cell-killing performance of Fe_3_O_4_ NPs is a function of cell endocytosis and enzyme-like activity (Fu et al., 2017).

### Metal NPs for Modulation TME ECM

Like any other tissue in the human body, the tumor has its own ECM, which serves as a supportive structure for tumor growth, migration, and metastasis. Collagens, elastin, fibronectins, laminins, glycoproteins, and proteoglycan are the common tumor ECM components. Therefore, ECM alteration is of great importance for TME modulation. The ECM modulation of tumors can be performed in various ways, including ECM disruption that mimics the tumor ECM to obstruct tumor progression, and intrusion in native ECM construction. Multiple methods are used for ECM elimination, including physical processes such as photothermal, hyperthermia, ultrasound, biochemical enzymes, and chemical agents (Chen et al., 2018). For example, Kolosnjaj-Tabi et al. have proposed a silica-coated iron oxide nanochain as an efficient, super magnetic NPs for ECM degradation of cancer tissue through PDT. The effect of fabricated metal-based nanochain was evaluated in the cancer model through near-infrared irradiation. According to the *in vitro* investigation, the cancer cells were eliminated, wherein the potency of this nanochain to melt the collagen matrix has been proposed (Kolosnjaj-Tabi et al., 2019).

Nevertheless, ECM degradation is an essential step in the metastasis process, where cancer cells need more space to be overproliferated, and ECM represents a substantial obstacle. Consequently, maintaining the tumor ECM or rebuilding it appears to be a logical procedure to overcome tumor cells in such a situation. Hu et al. proposed a transformable formulation as an artificial ECM for preventing the tumor metastasis. The primary mechanism of their proposed procedure depended on the NPs transformation into nanofibers. An RGD ligand-integrin receptor performed this transformation structure. The RGD binding process to integrins is significantly dependent on the RGD interactions metal ions such as Ca^2+^, Mg^2+^ at “metal ion-dependent adhesion site” (MIDAS) (Hu et al., 2017).

## Strategies for Metal NPs to Modulate Immune Responses

Instead of killing cancer cells directly, Metal NPs mainly modulate immune organs or immune cells to eradicate cancer cells. By injecting into the tumor medium, Metal NPs activate APCs to improve antigen delivery and T cell immune responses. They also increase antitumor efficacy by stimulating the immune system *in situ* and regulating T cell viability. Cytokines engineered into NPs can be transported to TME to increase antitumor activity (Liu et al., 2014).

Lymph node dendritic cells (DCs) are vital cells for processing and delivering antigens. The results showed that by attaching the nanoparticles to these cells, the nanoparticles could be directed to the specified TME. Because these immune cells are constantly delivering antigens to the tumor environment, transfecting them into tumor tissue can increase T lymphocytes and lead to more cancer cell death (Liu et al., 2014; Wilson et al., 2019).

**FIGURE 5:**
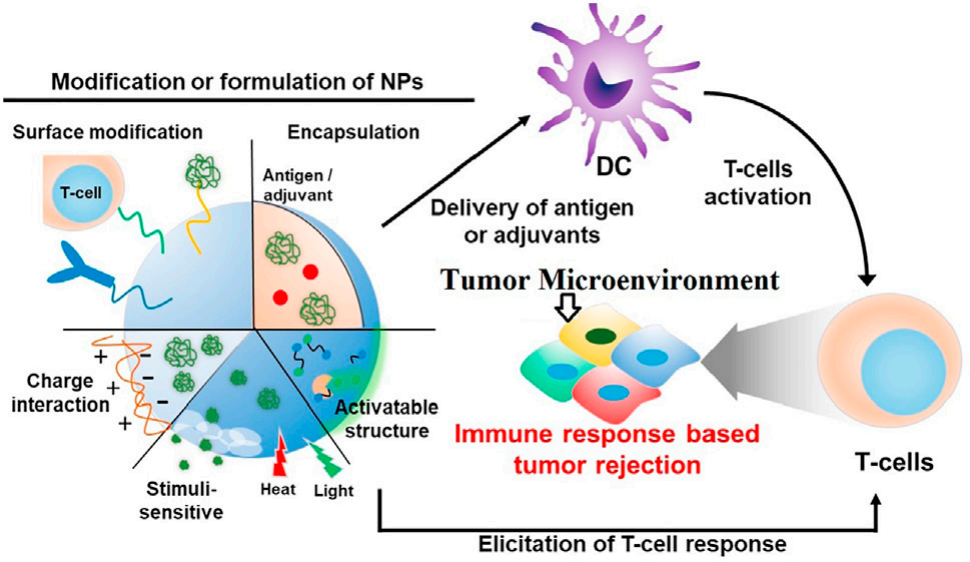
tumor antigen-specific T-lymphocytes for cancer immunotherapy. During cancer immunotherapy, after preparation and presentation of antigen by different molecular methods on dendritic cells, T cells are activated. Immune cells are one of the most important components. Activation of T cells is associated with the development of a specific immune response and destroys the tumor (Yoon et al., 2018).

The findings show that the binding of metal nanoparticles to immune cells can increase the effectiveness of cancer treatment and be used as anti-cancer vaccines in the future. Cancer vaccines use immune system mechanisms to identify tumor cells. In this way, cancer cells are detected by the immune system after antigenic changes and the progression of cancerous tissue is prevented (Li et al., 2018). However, after preparation and presentation of antigen by DCs, activation and proliferation of T cells is very important for cancer immunotherapy (Xin Yu et al., 2019; Li and Burgess, 2020; Zhu et al., 2020) (Figure 5).

The results show that different nanomaterials can be used as immune stimulants to activate T cells in TME (Stephan et al., 2010; Park et al., 2012; Schmid et al., 2017). Poly (lactic-co-glycolic acid) (PLGA) is a nanomaterial that binds to drug nanoparticles and targets T cells in TME to activate these cells and eradicate tumor cells. In most cases, nanomaterials are attached to nanoparticles by encapsulation (Zheng et al., 2013; Tang et al., 2018; Wang et al., 2018).

Various molecular mechanisms have been proposed to increase the effectiveness of this method, and in short, all of these strategies are based on increasing the death of tumor cells by the immune system. In fact, by this mechanism, it detects and destroys the specific immunity of cancer cells, thus reducing the inflammatory response in TME (Francis and Thomas, 2017; Meir et al., 2017; Smith et al., 2017).

## Metallic Nanoparticles and Clinical Effects of Cancer

Various studies have shown the role of different nanoparticles on cancer inhibition (Table 1). In one study, the anti-cancer effect of an organic metal nanoparticle was investigated. These findings showed the stability of nanoparticles and its effect on reducing tumor growth was significant. This was the first report to use MOF-derived nanoparticles in targeted nuclear PDT (Zeng et al., 2020). In another study, MOF-derived nanoparticles were used to alter cellular redox homeostasis. The findings show the high potency of these nanoparticles in improving the anti-cancer performance of PDT and suggest a new way to increase the therapeutic power based on ROS (Cheng et al., 2019).

**TABLE 1:** Metallic nanoparticles for the modulation of tumor microenvironment.

Result	Sample	Type of nanoparticle	Type of study	Running title	Author/Year
Au nanorods (NRs), Au nanoshells, other Au-related nanomaterials, graphene oxide, upconversion nanoparticles, and other related materials [including materials such as CuS, Fe_3_O_4_-related systems, and carbon nanotubes (CNTs)] proposed as good NIR nanomaterials	Cell lines	Near-infrared light-responsive (NIR) nanomaterials	review	Near-infrared light-responsive nanomaterials in cancer therapeutics	Shanmugam et al. (2014a)
The *in vitro* and *in vivo* results demonstrate that this platform selectively delivers anti-cancer drugs to target cells, releases them upon NIR irradiation, and effectively inhibits tumor growth through thermo-chemothera	Tumor growth in a mouse model	near-infrared (NIR): complementary DNA strands, the gold NR (50 nm × 10 nm), and a polyethylene glycol (PEG) layer	Animal	DNA Self-Assembly of Targeted Near-Infrared-Responsive Gold Nanoparticles for Cancer Thermo-Chemotherapy†	Xiao et al. (2012b)
This targeting vehicle provided remote-controlled delivery of this high toxic cargo cocktail at the tumor site, ensuring extra specificity that can avoid acute toxicity, where release of Dox and Pt (IV) was achieved upon NIR 808 nm diode laser irradiation	Tumor growth in a mouse model	Au nanorods (NRs)	Animal	Oligonucleotides—Assembled Au Nanorod-Assisted Cancer Photothermal Ablation and Combination Chemotherapy with Targeted Dual-Drug Delivery of Doxorubicin and Cisplatin Prodrug	Shanmugam et al. (2014b)
Rod-in-shell structure was a promising hyperthermia agent for the *in vivo* photothermal ablation of solid tumors when activated using a continuous-wave 808 m (first NIR window) or a 1,064 nm (second NIR window) diode laser	Tumor growth in a mouse model	Au nanorod (NR)	Animal	Au Nanorod Design as Light-Absorber in the First and Second Biological Near-Infrared Windows for *in Vivo* Photothermal Therapy	Tsai et al. (2013)
Multifunctional nanoparticle composed of a single, amine-modified gold nanorod, decorated with multiple “pearls” of Fe3O4 nanoparticles capped with carboxy groups showed simultaneous targeting, dual-mode imaging, and photothermal ablation of breast cancer cells is demonstrated	Breast cancer cells	Gold Nanorod/Fe3O4 Nanoparticle	*In vitro*		Wang et al. (2009)
The multifunctional APS/AuNR/PLGA-PEG nanoparticles can serve as an excellent synergistic agent for Focused ultrasound (FUS) therapy, facilitating real-time imaging, promoting thermal ablation effects, and boosting FUS-induced immune effects	Tumor growth in a mouse model	EGylated PLGA nanoparticles encapsulating astragalus polysaccharides (APS) and gold nanorods (AuNRs)	*In vitro* and *in vivo*	Multifunctional Nanoparticles Encapsulating Astragalus Polysaccharide and Gold Nanorods in Combination with Focused Ultrasound for the Treatment of Breast Cancer	Xiong et al. (2020)
The efficient phagocytosis of Au nanoshells by both monocytes and macrophages, photoinduced ablation of Au-nanoshellladen monocytes/macrophage, tumor recruitment, and photoinduced cell death of macrophages in the hypoxic microenvironment of a human breast tumor spheroid have all been successfully demonstrated	Human breast tumor spheroids	Au nanoshells	*In vitro*	A Cellular Trojan Horse for Delivery of Therapeutic Nanoparticles into Tumors	Choi et al. (2007)
Cancer cells targeted with the MagGNS AbHER2/neu *in vitro* were detectable by a commercial clinical MRI system, and were rapidly destroyed upon short exposure to femtosecond laser pulses with an NIR wave-length and a low power	SKBR3 cells	Magnetic gold nanoshells (Mag-GNS)	*In vitro*	Designed Fabrication of Multifunctional Magnetic Gold Nanoshells and Their Application to Magnetic Resonance Imaging and Photothermal Therapy	Kim et al. (2006)
HeLa cells incubated with GNS-MCs *in vitro* can be killed photothermally by exposure to NIR light	HeLa cells	Novel multifunctional theranostic agent based on gold-nanoshelled microcapsules (GNS-MCs)	*In vitro*	Gold-Nanoshelled Microcapsules: A Theranostic Agent for Ultrasound Contrast Imaging and Photothermal Therapy	Ke et al. (2011)
The Aptamer AS1411 show excellent stability. Significantly, the Mn3O4-PEG @ C & A inhibited tumor growth in a high-performance mouse model without any biotoxicity	Tumor growth in a mouse model	A new nanoenzyme (Mn3O4-PEG @ C & A) with the inherent activity of catalase	Animal Clinical	nanoenzyme for enhancing nucleus-targeted photodynamic therapy	Zeng et al. (2020)
Inside tumor cells can effectively block the Rx removal pathway mediated	Liver tumor cells	A porous metal-organic (MOF) framework as a photodynamic therapy agent (PDT) and a transporter for the alkaloid transporter piperlongumin (PL)	Animal Clinical	Nanotherapeutics interfere for highly photodynamic therapy	Cheng et al. (2019)
PDT and TrxR inhibition causes a profound increase in cellular ROS levels					
Within 1 h, doxorubicin could reach its destination, DNA, in the nucleus without degradation, while PLGA nanoparticles, were still in the chamber and lysosomes were observed	Brain tumor cells	doxorubicin-loaded PLGA nanoparticles	Human clinical	Delivery of nanoparticles into glioblastoma cells	Malinovskaya et al. (2017)
Significant antitumor effect of doxorubicin nanoparticles was observed. PLGA-coated poloxamer nanoparticles with doxorubicin transport through are effective in the treatment of glioblastoma	Tumor cells in mice	Poly (lactic-co-glycolic acid) (PLGA) nanoparticles	Animal clinical	Efficient Chemotherapy Using Nanoparticles with Different Stabilizers	Wohlfart et al. (2011)
This nanoparticle is able to improve the therapeutic index. The strong anti-cancer activity of this nanomedicine is promising. The strong anti-cancer activity of this nanomedicine is promising	lung cancer cell line, liver cancer cell line and Breast cancer cell line	Copper oxide nanoparticles (CuO NPs)	Human clinical	copper oxide nanoparticles for augmenting anticancer activity	Abu-Serie and Eltarahony (2021)
Combining the natural alkaloid Ber with C60 could be a new treatment strategy for lung cancer	LLC cells in mice	Berberine (Ber) combined with C60	Human clinical	Antitumor efficiency of the alkaloid complexed with C60 fullerene in Lewis lung carcinoma	Grebinyk et al. (2021)
Unlike Nanoparticles, showed an inhibitory effect on the expression of genes encoding the NLRP3 inflammatory complex, but also reduced activation of the NLRP3 inflammatory complex. The combination of gallic acid with CSNP suppressed the immune system in cervical cancer	Cervical cancer cell lines	Nanoparticles (CSNP) and gallic acid conjugated gallic acid (gCSNP)		Nanoparticles modulates NLRP3 inflammasome complex activation in cervical cancer	Chakraborty et al. (2021)
Gold nanorods have been specifically mentioned as a new agent for simultaneous bioimaging and cancer treatment	Tumor cell lines in breast cancer	Gold Nanorods (GNRs)	review	Synthesis of gold nanorods and photothermal therapy	Khan et al. (2021)
Strong immune responses at extracellular CDN concentrations are less than 100-fold *in vitro*. The formulation of CDBA PBAE nanoparticles improves potency in the treatment of melanoma	Melanoma tumors B16	Polybeta amino ester (PBAE) nanoparticles to deliver CDN to the cytosol	Clinical	nanoparticles for enhanced cancer immunotherapy	Wilson et al. (2018)

Other studies have shown the effect of PLGA encapsulation in docorbiocin on immune stimulation (Wohlfart et al., 2011; Malinovskaya et al., 2017). The findings of this study confirm the innovation in immunotherapy methods with the help of metal nanoparticles and suggest different methods to increase the efficiency and cost-effectiveness of treatment (Abu-Serie and Eltarahony, 2021; Grebinyk et al., 2021). Findings on the effect of CSNP nanoparticles on inhibiting the growth of uterine cancer cells showed the effectiveness of this nanoparticle. The researchers stated that future research could examine the CSNP-modulating immune mechanism as potential treatment strategies aimed at escaping immunity as an important feature of cancer (Chakraborty et al., 2021). Another study showed that PBAE nanoparticles in the nanoparticle-mediated cytosolic delivery method for STING agonists synergize with cell cycle inhibitors, and this synergy has a strong potential to enhance cancer immunotherapy (Wilson et al., 2018).

## Conclusion

The distinctive features of TME provide the opportunity to exploit its use as a new approach to cancer treatment. The high metabolism of cancer cells and the excessive craving for proliferation prevents the development of new vascular tissues and vessels, and this in itself can be used in the successful delivery of therapeutic agents to these cells, which today are known as the EPR effect. Furthermore, due to the high metabolism of these cells, the tumor environment has high hypoxia conditions which can be utilized extensively in sonodynamic therapy. The use of metal nanoparticles as sonosensitizers addresses the problem of natural sonosensitizers, which have low solubility and viscosity, and as a result, ensuing ROS can destroy tumor tissue. Besides, the fantastic optical properties of metal nanoparticles, including gold nanoparticles, have received much attention in photodynamic therapy. By producing oxygen in cancerous tissues and using suitable radiation, photosynthesizers such as metal nanoparticles can create highly toxic ROS. The effect of acidic environment on TME has been discussed, and it was shown that systems designed with metal nanoparticles could use this low pH condition to release their drug and provide a high-impact combination therapy. At low pH, ferromagnetic nanoparticles kill cancer tissue by converting H_2_O_2_ to toxic singlet O_2_ species. Overall, we see special consideration to metal nanoparticles. Given the tremendous potential metal nanoparticles have resemblance in TME modulation, there looks to be a promising future for cancer therapy. The most critical challenges to be considered in future research are targeting and toxicity, which should be carefully considered. In addition to the above, it is important to consider new therapeutic strategies for the use of metal nanoparticles in immunotherapy. Because despite the progress, many efforts are still needed to apply cancer treatment with minimal side effects. Barriers to biological research must be removed. Then, the necessary conditions for clinical research will be provided so that in the future, like conventional treatments, nanoparticles can be taken as an effective step to reduce the problems of cancer patients.
